# Belinostat in combination with standard cyclophosphamide, doxorubicin, vincristine and prednisone as first-line treatment for patients with newly diagnosed peripheral T-cell lymphoma

**DOI:** 10.1186/s40164-021-00203-8

**Published:** 2021-02-18

**Authors:** Patrick B. Johnston, Amanda F. Cashen, Petros G. Nikolinakos, Anne W. Beaven, Stefan Klaus Barta, Gajanan Bhat, Steven J. Hasal, Sven De Vos, Yasuhiro Oki, Changchun Deng, Francine M. Foss

**Affiliations:** 1 Mayo Clinic, 200 first St SW, Rochester, MN 55905 USA; 2 Division of Oncology, Washington University Medical School, 660 S. Euclid Ave, Campus, Box 8007, St Louis, MO 63110 USA; 3 University Cancer and Blood Center, 3320 Old Jefferson Rd #700, Athens, GA 30607 USA; 4 Duke University School of Medicine, 2592 Morris Bldg, Box 3406, Durham, NC 27710 USA; 5 Dept of Hematology/Oncology, Fox Chase Cancer Center, 333 Cottman Ave, Philadelphia, PA 19111 USA; 6 Spectrum Pharmaceuticals, 157 Technology Dr, Irvine, CA 92618 USA; 7 Cancer Care, Ronald Reagan University of California At Los Angeles Medical Center, 2020 Santa Monica Blvd, Santa Monica, CA 90404 USA; 8 Dept of Lymphoma/Myeloma, University of Texas MD Anderson Cancer Center, 1515 Holcombe Blvd, Unit 0429, Houston, TX 77030 USA; 9 Center for Lymphoid Malignancies, Columbia University Medical Center, 51 West 51st St, New York, NY 10019 USA; 10 Medical Oncology, Yale Cancer Center, 333 Cedar St, TMP 3, PO Box 208028, New Haven, CT 06510 USA

**Keywords:** Peripheral T-cell lymphoma (PTCL), Histone deacetylase inhibitor (HDACi), Belinostat, CHOP

## Abstract

**Background:**

Belinostat is a histone deacetylase inhibitor approved for relapsed refractory peripheral T-cell lymphoma (PTCL). The primary objective of this study was to determine the maximum tolerated dose (MTD) of belinostat combined with CHOP (Bel-CHOP). Secondary objectives included safety/tolerability, overall response rate (ORR), and belinostat pharmacokinetics (PK).

**Methods:**

Patients were ≥ 18 years with histologically confirmed, previously untreated PTCL. Patients received belinostat (1000 mg/m^2^ once daily) + standard CHOP for 6 cycles with varying schedules using a 3 + 3 design in Part A. Part B enrolled patients at MTD dose.

**Results:**

Twenty-three patients were treated. One patient experienced DLT (Grade 3 non-hematologic toxicity) on Day 1–3 schedule, resulting in escalation to Day 1–5 schedule (n = 3). No DLTs were observed and Day 1–5 schedule with 1000 mg/m^2^ was declared as MTD. Twelve additional patients were enrolled in Part B using MTD. Median relative dose intensity was 98%. All patients experienced adverse events (AEs), including nausea (78%), fatigue (61%), and vomiting (57%). Serious AEs occurred in 43%, with febrile neutropenia (17%) and pyrexia (13%). Overall ORR was 86% with 71% reported CR at MTD. Belinostat PK parameters were similar to single-agent.

**Conclusions:**

Bel-CHOP was well tolerated and MTD in CHOP combination was the same dose and schedule as single agent dosing.

*Trial Registration*: ClinicalTrials.gov Identifier: NCT01839097.

## Background

Peripheral T-cell lymphoma (PTCL) refers to a heterogeneous group of mature T-cell and natural killer (NK)-cell aggressive non-Hodgkin’s lymphomas (NHLs) accounting for 10–15% of all newly diagnosed NHLs [1–3]. According to the World Health Organization (WHO) classification, mature T-cell and NK-cell neoplasms are classified according to a combination of morphologic, immunophenotypic, genetic, and clinical features into 22 distinct entities, the most common of which include PTCL not otherwise specified (PTCL-NOS), angioimmunoblastic T-cell lymphoma (AITL), and anaplastic large-cell lymphoma (ALCL), which collectively represent 66% of all cases of PTCL in North America [4, 5]. The median overall survival (OS) of PTCL is low (< 2 years), with a reported 5-year survival of up to 33% [2, 5–8].

First-line treatment of PTCL often comprises combination therapy with cyclophosphamide, doxorubicin, vincristine, and prednisone (CHOP). Despite its widespread use, CHOP has not been studied in prospective, randomized studies and was associated with a dismal 5-year OS of only 37% in a retrospective meta-analysis of 2,912 PTCL patients treated with CHOP or CHOP-like regimens [9–11]. However, no other single-agent or combination regimen has demonstrated superior efficacy to CHOP and it therefore remains a primary choice for first-line therapy for PTCL. In an attempt to increase the response rate and improve the durability of responses, several modifications to the CHOP regimen have been investigated with no clear improvements in long-term group efficacy observed, including the addition of agents (e.g., bleomycin, gemcitabine, etoposide, vindesine, liposomal doxorubicin, and bevacizumab), administration of more intensive dosing, and the use of autologous stem cell rescue as consolidation therapy in patients who attain complete remissions from high-dose chemotherapy [12–16]. Allogeneic stem cell transplantation has also been found to be feasible in the subset of patients with PTCL who are candidates for the procedure, but it has been associated with significant treatment-related toxicity [17, 18].

Since 2009, several novel agents have been approved in the United States (US) specifically for the treatment of PTCL in the relapsed/refractory setting, including pralatrexate (a rationally designed antifolate), brentuximab vedotin (an antibody drug conjugate), and the histone deacetylase inhibitors (HDACi) romidepsin and belinostat [19–22]. Romidepsin was recently investigated in a dose-finding combination study with CHOP in patients with previously untreated PTCL [23]. Results from this study indicated a 69% overall response rate (ORR), median progression-free survival (PFS) of 21.3 months, OS of 71% at 30 months, and a toxicity profile that was largely reflective of the manageable hematological and gastrointestinal events associated with the individual drugs constituting the regimen. Similarly, an HDACi used to treat cutaneous T-cell lymphoma (CTCL), vorinostat, was combined with CHOP in previously untreated patients with PTCL, resulting in an ORR of 93%, median duration of response (DoR) of 29 months, median PFS of 31 months, and 2-year PFS and OS rates of 79 and 81%, with no notable cumulative toxicity observed [24].

The current Phase 1 study was conducted to establish the MTD of the HDACi belinostat when combined with CHOP (Bel-CHOP) in previously untreated PTCL. Belinostat monotherapy demonstrated activity in patients with relapsed/refractory PTCL in a Phase 2 registration study, in which the ORR was 26% in 120 evaluable patients, with 61% of responders achieving their response within 30 to 45 days of the first dose and a median DoR of 13.6 months [22]. In addition to the positive results observed with other HDACi plus CHOP studies [23], the rationale for Bel-CHOP therapy was based on the anticipated synergistic effect of the regimen, as belinostat and each of the components of the CHOP regimen target different aspects of the cell cycle with unique mechanisms of action [25]. In addition, preclinical studies using PTCL cell lines (KARPAS-299 and SR-299) demonstrated synergistic antitumor activity of belinostat combined with cyclophosphamide, doxorubicin, and vincristine. Belinostat plus dexamethasone demonstrated modest activity and no safety concerns were observed in a Phase 2 study in patients with multiple myeloma [25]. Furthermore, as each of the agents in the Bel-CHOP regimen exhibits a different metabolic safety profile, overlapping toxicity was anticipated to be minimal.

## Methods

### Study design and treatment

This 2-part, open-label Phase 1 dose-finding study was designed to determine the maximum tolerated dose (MTD) of Belinostat when administered in combination with standard CHOP therapy (Bel-CHOP) comprising cyclophosphamide (750 mg/m^2^ intravenously [IV] on Day 1), doxorubicin (50 mg/m^2^ IV on Day 1), vincristine (1.4 mg/m^2^ [maximum 2 mg] IV on Day 1), and prednisone (100 mg orally on Days 1–5) in up to 6 continuous 21-day cycles in patients with newly diagnosed PTCL who were candidates for first-line chemotherapy with CHOP. In Part A, a traditional 3 + 3 dose-escalation schema was implemented, in which 2 sequential dose schedule cohorts were enrolled to determine the MTD of Bel-CHOP. Up to 6 evaluable patients were to be assessed for dose-limiting toxicities (DLT) in each cohort during Cycle 1 to inform dose-escalation/reduction decisions. In all cohorts, patients received standard CHOP therapy in up to 6 continuous 21-day cycles. Belinostat treatment comprised 1000 mg/m^2^ administered IV infusion over 30 min, with the initial cohort (Cohort 3) receiving belinostat on Days 1–3 of every cycle and subsequent cohorts receiving belinostat for an increased or decreased number of days based on observed toxicity. The maximum administered dose (MAD) (Cohort 5) was not to exceed the single-agent dosing schedule of belinostat (1000 mg/m^2^ of belinostat administered on Days 1–5). In Part B, the MTD/MAD, as determined in Part A, was to be evaluated in 10 additional patients to further define the safety and tolerability and to establish the recommended Phase 3 dose (RP3D) of belinostat in the Bel-CHOP regimen. Belinostat was administered 15 ± 5 min prior to CHOP on days of co-administration. Prophylactic antiemetics and granulocyte colony-stimulating factor (G-CSF) were also administered per the 2006 American Society of Clinical Oncology (ASCO) Guidelines. Treatment was administered for a maximum of 6 cycles or until death, progressive disease (PD), unacceptable toxicity, substantial noncompliance, initiation of a new anticancer therapy, or patient, investigator, or Sponsor decision to withdraw, whichever comes first. Belinostat dose reductions due to toxicity were permitted after Cycle 1, including a reduction in the number of days of administration and/or in the dosage (to 750 or 500 mg/m^2^).

The primary objective was to determine the MTD and RP3D of belinostat in the Bel-CHOP regimen. Secondary objectives included evaluation of the safety and tolerability of combination treatment, the overall response rate (ORR), and the pharmacokinetics (PK) of belinostat when co-administered with CHOP during Part A.

The study protocol and patient materials were approved by institutional review boards and/or ethics committees at all sites. Study conduct followed International Conference on Harmonization (ICH) Guidelines for Good Clinical Practice, including written informed consent and monitoring of all data.

### Patients

Eligible patients were ≥ 18 years of age with histologically confirmed PTCL or transformed CTCL, an Eastern Cooperative Oncology Group (ECOG) performance status of ≤ 2, and a life expectancy of ≥ 3 months. Patients must have had measurable disease based on Cheson criteria [26] and been eligible for first-line CHOP therapy. Patients must have had adequate hematologic, hepatic, and renal function, including absolute neutrophil count (ANC) ≥ 1.5 × 10^9^, platelets ≥ 100,000/mm^3^, alanine and aspartate aminotransferases (AST and ALT) ≤ 3 × the upper limit of normal (ULN), total bilirubin ≤ 2.0 mg/dL, calculated creatinine clearance ≥ 50 mL/min [27], and prothrombin time or international normalized ratio < 1.5 × ULN (or in the therapeutic range of anticoagulation therapy). Prohibited prior therapy included HDAC inhibitors (except for CTCL), extensive radiotherapy, severely myelotoxic regimens, and stem cell transplantation. Patients with ≥ Grade 3 neuropathy, cardiovascular disease (including prolonged QT), and active infections requiring therapy were also excluded.

### Study assessments

Safety assessments included physical examinations, adverse event (AE) monitoring, electrocardiograms (ECGs), and changes in laboratory parameters. AEs were graded using the National Cancer Institute (NCI) Common Terminology Criteria for Adverse Events (CTCAE), Version 4.03. AE causality was attributed by Investigators to combined study treatment rather than to individual regimen components. During treatment, ECGs were performed prior to and 1 h after belinostat infusion on Day 1 of each cycle and Days 4 and 5 for patients in Cohort 5. After discontinuing therapy, patients completed an End of Study (EOS) Visit 30 days after their last dose of study treatment.

Radiographic tumor assessments by each study center were conducted using computed tomography, positron emission tomography, or magnetic resonance imaging according to the International Harmonization Project (IHP) revision of the International Working Group criteria [26]. Assessments were performed at baseline, every 2 cycles (6 weeks) thereafter, and at the EOS. Patients with baseline bone marrow assessments that were positive for lymphoma were required to have a repeat bone marrow biopsy/aspirate for confirmation of a complete response (CR).

During Part A, PK samples for the determination of plasma concentrations of belinostat and its 5 major metabolites were collected on Cycle 1 Day 2 pre-infusion and at 8 post-infusion time points through 7.5 h after the start of infusion. Plasma concentrations were determined using a validated liquid chromatography-tandem mass spectroscopy method (Covance Laboratories, Madison, Wisconsin).

### Statistical analysis

The total sample size planned for this study was up to 28 evaluable patients distributed in three cohorts of up to six patients each and 10 additional patients in dose expansion phase. The DLT rate and the corresponding 90% confidence intervals for 1 and 2 DLTs in each dose cohorts are 16.7% (0.9%, 58.2%) and 33.3% (6.3%, 72.9%) respectively.

All treated patients were included in the Safety Population, and patients who completed Cycle 1 were evaluable for DLTs, which included the following toxicities occurring during Cycle 1:

1. Any ≥ Grade 3 nonhematological toxicity.
2. Platelet count of 25 × 10^9^/L for ≥ 7 days or platelet nadir 10 × 10^9^/L at any time.
3. Failure to recover platelet count ≥ 75 × 10^9^/L and/or ANC ≥ 1.5 × 10^9^/L by Day 28.
4. ANC < 0.5 × 10^9^/L lasting for ≥ 7 days despite G-CSF administration.
5. Treatment toxicity requiring dose reduction in Cycle 2.
6. Nonhematological toxicity requiring a delay in Cycle 2 for > 7 days.

## Results

### Patient disposition and baseline characteristics

**Fig. 1:**
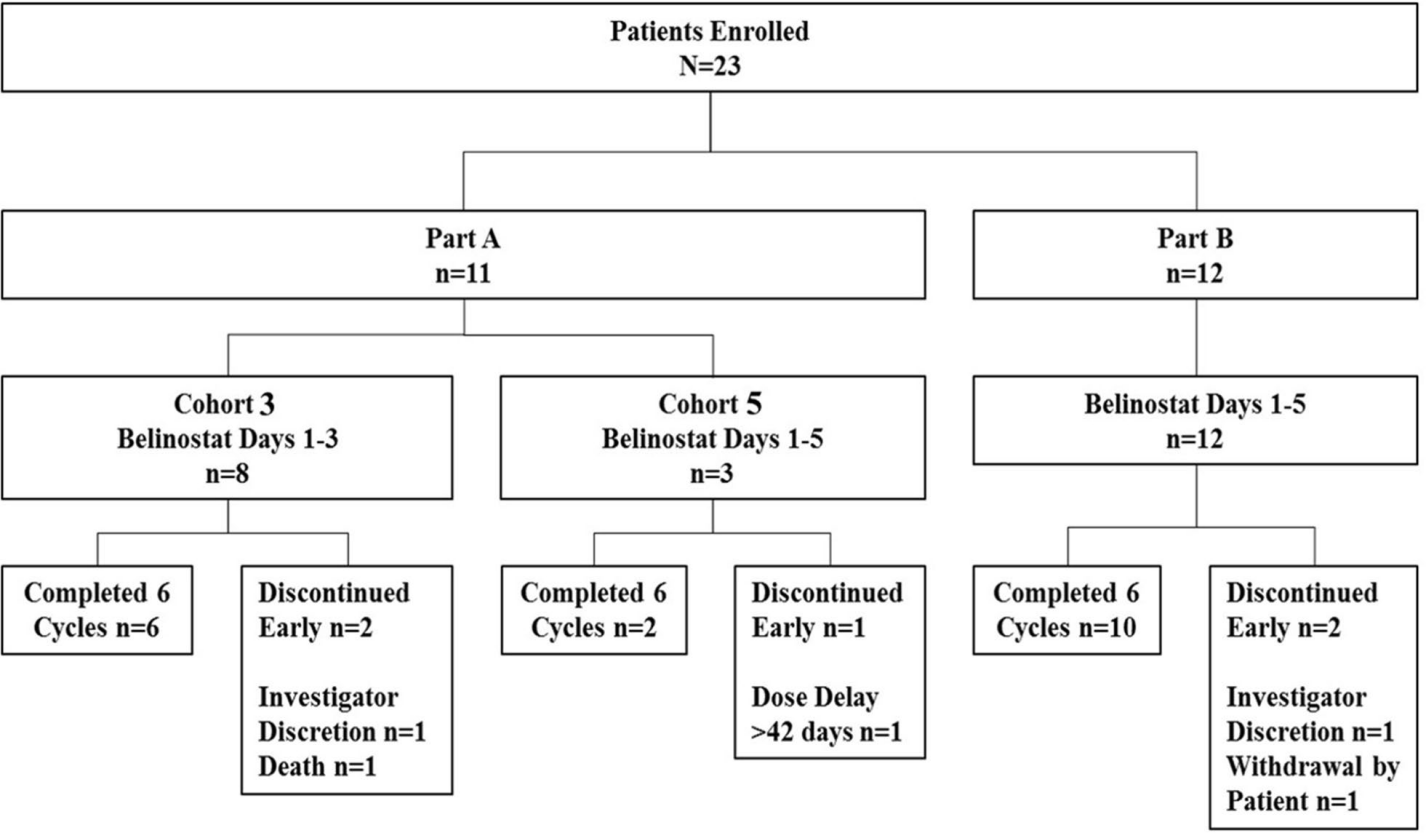
Patient enrollment and disposition

Between August 2013 and August 2015, 23 patients with PTCL were enrolled and treated at 9 investigational sites in the US, including 11 patients in Part A and 12 patients in Part B (Fig. 1). Although the initial plan had been to enroll 10 patients in Part B, one patient was replaced due to a patient discontinuing early, and the additional patient was enrolled because two patients signed consent the same day to fill the last spot and the sponsor agreed to allow one extra patient to be included. Most patients (78%) completed 6 cycles of study treatment, with 5 (22%) patients discontinuing: 2 patients due to investigator decision, 1 because of the patient’s decision, 1 due to a treatment delay of > 42 days, and 1 death due to respiratory failure.

The majority of patients were male (65%), White (65%) and the median age was 63.0 (range 35–84) years (Table 1). The majority had baseline disease stage of IVA and IVB (61%) with ECOG performance status of 0 (39%) or 1 (52%).

**Table 1:** Baseline patient characteristics—safety population (N = 23)

Parameter		Cohort 3 (Bel D1-3) n = 8	Cohort 5 + Expansion (Bel D1-5) n = 15	Total N = 23
Age (Years)	Mean ± SD	61.9 ± 13.75	62.5 ± 8.91	62.3 ± 10.52
	Median (range)	62.5 (35, 84)	63.0 (44, 77)	63.0 (35, 84)
Gender, n (%)	Female	4 (50)	4 (27)	8 (35)
	Male	4 (50)	11 (73)	15 (65)
Race, n (%)	White	6 (75	9 (60)	15 (65)
	Black or African American	2 (25)	4 (27)	6 (26)
	Latino	0	1 (7)	1 (4)
	Other	0	1 (7)	1 (4)
ECOG Performance Status, n (%)	0	3 (38)	6 (40)	9 (39)
	1	5 (63)	7 (47)	12 (52)
	2	0	2 (13)	2 (9)
PTCL Subtype, n (%)	ALCL, ALK-negative	1 (13)	0	1 (4)
	ALCL, ALK-positive	1 (13)	1 (7)	2 (9)
	Angioimmunoblastic TCL	1 (13)	9 (60)	10 (43)
	Cytotoxic T-cell phenotype	0	1 (7)	1 (4)
	PTCL NOS	5 (63)	4 (27)	9 (39)
Disease Stage, n (%)	IIB	0	2 (13)	2 (9)
	IIIA	3 (38)	2 (13)	5 (22)
	IIIB	0	2 (13)	2 (9)
	IVA	1 (13)	5 (33)	6 (26)
	IV B	4 (50)	4 (27)	8 (35)
International Prognostic Index	0—Low Risk	0 (0)	1 (7)	1 (4)
	1—Low-Intermediate Risk	3 (38)	3 (20)	6 (26)
	2—High-Intermediate Risk	4 (50)	7 (47)	11 (48)
	3—High Risk	1 (13)	4 (27)	5 (22)

*ALCL *anaplastic large cell lymphoma, *ALK *anaplastic lymphoma kinase, *Bel *belinostat, *D *day, *ECOG* Eastern Cooperative Oncology Group, *n* or *N* number, *NOS* not otherwise specified, *PTCL* peripheral T-cell lymphoma, *SD* standard deviation, *TCL* T-cell lymphoma

### Determination of maximum tolerated dose

All 23 patients received CHOP + 1000 mg/m^2^ QD of belinostat, with a starting schedule of belinostat administered on Days 1–3 in Part A Cohort 3. One patient experienced DLT (Grade 3 nausea, vomiting, diarrhea and dehydration) and the cohort was expanded to 6 patients. One patient death occurred prior to evaluation and one patient made the decision not to participate in the clinical trial before receiving any chemotherapy. The patient in Cohort 3 who died was non-evaluable for determination of the MTD due to non-compliance with the required use of G-CSF per the ASCO Guidelines, but continued treatment; a total of 8 patients were treated in Cohort 3. Since only one of 8 patients (13%) in Cohort 3 experienced DLTs, the study was escalated to Cohort 5 (belinostat administered on Days 1–5). No DLTs were observed in 3 patients treated in Cohort 5; therefore, there was no need for cohort expansion and no planned dose escalation beyond Cohort 5, thus this dosing schedule was deemed the MTD. Based on the results for Cohorts 3 and 5, no patients were enrolled into Cohorts 1, 2, or 4. In Part B, 12 patients were treated at the MTD.

### Safety

The Bel-CHOP regimen was well tolerated in this study with most patients remaining at the target belinostat and CHOP doses for the total planned duration of 6 treatment cycles (Table 2). The median relative dose intensity was 98% at both belinostat dose levels. Belinostat dose modifications were required for 5 patients (22%) due to AEs. In Cohort 3, belinostat was delayed for 7 days after Cycle 5 due to hospitalization for febrile neutropenia (n = 1), and interrupted on Cycle 1 Day 2 due to Grade 1 nausea/vomiting (n = 1) and Grade 3 nausea/vomiting (n = 1). At the belinostat MTD, belinostat was interrupted in Cycle 1 due to Grade 1 nausea and vomiting (n = 1) and a Grade 2 belinostat infusion-related reaction (n = 1).

**Table 2:** Treatment Exposure, Modifications, and Dose-limiting Toxicity – Safety Population (N = 23)

Parameter (unit)	Cohort 3 (Bel D1-3) n = 8^a^	Cohort 5 + Expansion (Bel D1-5) n = 15
Number of Bel-CHOP cycles administered, median (range)	6.0 (1–6)	6.0 (1–6)
Cumulative dose received, median (range)		
Belinostat (mg/m^2^)	17,622.0 (3036–18,000)	29,120.0 (4815–30,510)
Cyclophosphamide (mg/m^2^)	4421.5 (761–4506)	4460.0 (720–4578)
Doxorubicin (mg/m^2^)	295.0 (50–300)	297.0 (49–306)
Vincristine (mg)	12.0 (2–12)	12.0 (2–12)
Prednisone (mg)	3000.0 (300–3000)	3000.0 (500–3000)
RDI (%), median (range)		
Belinostat	98.5 (94–101)	98.0 (92–102)
Cyclophosphamide	99.5 (84–101)	99.0 (92–102)
Doxorubicin	99.0 (85–100)	100.0 (92–102)
Vincristine	100.0 (75–100)	100.0 (75–00)
Prednisone	100.0 (60–100)	100.0 (93–100)
Patients with dose reduction due to AE, n (%)		
Belinostat	1 (13)	0
Cyclophosphamide	2 (25)	0
Doxorubicin	2 (25)	0
Vincristine	1 (13)	1 (7)
Patients with dose interruption due to AE, n (%)		
Belinostat	2 (25)	2 (13)
Doxorubicin	0	1 (7)
Vincristine	1 (13)	0
Patients with dose delay due to AE, n (%)		
Belinostat	1 (13)	0
Cyclophosphamide	1 (13)	0
Doxorubicin	1 (13)	0
Vincristine	1 (13)	0
Prednisone	1 (13)	0
Patients with DLT in Cycle 1, n (%)	1 (13)	0
Grade 3 nausea, vomiting, diarrhea, and dehydration	1 (13)	0

*AE* adverse event, *DLT* dose-limiting toxicity, *n* number, *RDI* relative dose intensity

^a^1 patient did not complete Cycle 1 and therefore was not evaluable for DLT assessment

All patients experienced at least one treatment-emergent adverse event (TEAE), with the most commonly reported events including nausea (78%), fatigue (61%), and vomiting (57%; Table 3). TEAEs were considered related to Bel-CHOP therapy for 96% of patients and most frequently included nausea (70%), fatigue and vomiting (each 57%), and anemia, constipation, and diarrhea (each 35%). Grade 3/4 treatment-related TEAEs were reported in 57% of patients. The most frequently reported Grade 3 or 4 TEAEs were hematological in nature, which is consistent with reported AEs observed with cytotoxic therapy.

**Table 3:** Treatment-emergent adverse events by maximum NCI CTCAE severity—safety population (N = 23)

MedDRA Preferred Term	Cohort 3 (Bel D1-3) n = 8 n (%)			Cohort 5 + Expansion (Bel D1-5) n = 15 n (%)			Total N = 23 n (%)		
	All Grades	Grade 1–2	Grade 3–4	All Grades	Grade 1–2	Grade 3–4	All Grades	Grade 1–2	Grade 3–4
All AEs (> 25% of pts)	8 (100)	0	8 (100)	15 (100)	5 (33)	10 (67)	23 (100)	5 (22)	18 (78)
Nausea	6 (75)	6 (75)	1 (13)	12 (80)	12 (80)	1 (7)	18 (78)	18 (78)	2 (9)
Fatigue	7 (88)	7 (88)	0	7 (47)	7 (47)	0	14 (61)	14 (61)	0
Vomiting	4 (50)	4 (50)	0	9 (60)	9 (60)	0	13 (57)	13 (57)	0
Anemia	4 (50)	3 (38)	2 (25)	5 (33)	4 (27)	3 (20)	9 (39)	7 (30)	5 (22)
Constipation	2 (25)	2 (25)	0	7 (47)	7 (47)	0	9 (39)	9 39)	0
Diarrhea	4 (50)	3 (38)	0	5 (33)	5 (33)	0	9 (39)	8 (35)	0
Alopecia	4 (50)	4 (50)	0	4 (27)	4 (27)	0	8 (35)	8 (35)	0
Decreased appetite	3 (38)	3 (38)	0	5 (33)	5 (33)	0	8 (35)	8 (35)	0
Dizziness	2 (25)	2 25)	0	6 (40)	6 (40)	0	8 (35)	8 (35)	0
Cough	3 (38)	3 (38)	0	4 (27)	4 (27)	0	7 (30)	7 (30)	0
Dysphonia	3 (38)	2 (25)	0	4 (27)	3 (20)	0	7 (30)	5 (22)	0
Neutrophil count decreased	3 (38)	1 (13)	3 (38)	4 (27)	1 (7)	4 (27)	7 (30)	2 (9)	7 (30)
Stomatitis	3 (38)	3 (38)	0	4 (27)	4 (27)	0	7 (30)	7 (30)	0
Upper respiratory tract infection	2 (25)	2 (25)	0	5 (33)	4 (27)	0	7 (30)	6 (26)	0
Headache	4 (50)	4 (50)	0	2 (13)	2 (13)	0	6 (26)	6 (26)	0
Neutropenia	3 (38)	1 (13)	3 (38)	3 (20)	1 (7)	3 (20)	6 (26)	2 (9)	6 (26)
Pruritus	2 (25)	1 (13)	0	4 (27)	0 (0)	0	6 (26)	1 (4)	0
Pyrexia	3 (38)	3 (38	0	3 (20)	3 (20)	0	6 (26)	6 (26)	0
WBC count decreased	2 (25)	0	2 (25)	4 (27)	2 13)	3 (20)	6 (26)	2 (9)	5 (22)
Related AEs (> 25% of pts)	7 (88)	2 (25)	5 (63)	15 (100)	7 (47)	8 (53)	22 (96)	9 (39)	13 (57)
Nausea	5 (63)	5 (63)	1 (13)	11 (73)	11 (73)	1 (7)	16 (70)	16 (70)	2 (9)
Fatigue	7 (88)	7 (88)	0	6 (40)	6 (40)	0	13 (57)	13 (57)	0
Vomiting	4 (50)	4 (50)	1 (13)	9 (60)	9 (60)	0	13 (57)	13 (57)	1 (4)
Anemia	3 (38)	2 (25)	2 (25)	5 (33)	4 (27)	2 (13)	8 (35)	6 (26)	4 (17)
Constipation	2 (25)	2 (25)	1 (13)	6 (40)	6 (40)	0	8 (35)	8 (35)	1 (4)
Diarrhea	4 (50)	3 (38)	1 (13)	4 (27)	4 (27)	0	8 (35)	7 (30)	1 (4)
Neutrophil count decreased	3 (38)	1 (13)	3 (38)	4 (27)	1 (7)	4 (27)	7 (30)	2 (9)	7 (30)
Decreased appetite	2 (25)	2 (25)	1 (13)	4 (27)	4 (27)	0	6 (26)	6 (26)	1 (4)
Stomatitis	3 (38)	3 (38)	0	3 (20)	3 (20)	0	6 (26)	6 (26)	0
SAEs (> 1 pt)^a^	3 (38)	2 (25)	2 (25)	7 (47)	2 (13)	7 (47)	10 (43)	4 (17)	9 (39)
Febrile neutropenia	1 (13)	1 (13)	1 (13)	3 (20)	0	3 (20)	4 (17)	0	4 (17)
Pyrexia	2 (25)	2 (25)	0	1 (7)	1 (7)	0	3 (13)	3 (13)	0
Nausea	1 (13)	0	1 (13)	1 (7)	0	1 (7)	2 (9)	0	1 (4)
Neutropenia	0	0	0	2 (13)	0	2 (13)	2 (9)	0	2 (9)
Discontinuation due to AE	1 (13)	0	1 (13)^b^	0	0	0	1 (4)	0	1 (4)^b^
Respiratory failure (fatal) secondary to PD	1 (13)	0	1 (13)^b^	0	0	0	1 (4)	0	1 (4)^b^

*AE* adverse event, *CTCAE* Common Terminology Criteria for Adverse Events, *MedDRA* Medical Dictionary for Regulatory Activities, *NCI* National Cancer Institute, *SAE* serious adverse event

^a^All SAEs reported in > 1 patient were assessed as related to study treatment

^b^Grade 5

Serious adverse events (SAEs) occurred in 43% of patients. SAEs included febrile neutropenia (17%), pyrexia (13%), and nausea and neutropenia (each 9%), all of which were considered related to Bel-CHOP study treatment.

One patient in Cohort 3 died on study days after the last scheduled dose of belinostat on Cycle 1, Day 15; the death was attributed to respiratory failure secondary to disease progression and was considered not related to Bel-CHOP study treatment as determined by the investigator. No other patients discontinued study treatment prematurely due to AEs.

### Efficacy

The Efficacy Population comprised 21 of the 23 treated patients; 2 patients discontinued treatment prior to undergoing the imaging studies for tumor response. In 18 of the 21 evaluable patients who completed 6 cycles of Bel-CHOP, the ORR was 86% in both Cohort 3 (6/7 patients) (CI: 42.1–99.6) and in Cohort 5 + expansion patients (12/14 patients) at the MTD (CI: 57.2–98.2) (Table 4). The rate of CR was 57% in Cohort 3 and 71% at the MTD (1000 mg/m^2^ belinostat for 5 days). Two patients in each belinostat cohort achieved a PR, 1 patient at the MTD maintained stable disease, and 1 patient at each dose level had PD. The ORR was similar across age groups, tumor subtypes, or if bone marrow lymphoma involvement or not. In particular, the ORR in AITL patients was 89% and it was 90% in patients with bone marrow involvement.

**Table 4:** Summary of overall best response—efficacy population (N = 21)

	Cohort 3 (Bel D1-3) n = 7	Cohort 5 + Expansion (Bel D1-5) n = 14
Overall best response, n (%)		
CR	4 (57)	10 (71)
PR	2 (29)	2 (14)
SD	0	1 (7)
PD	1 (14)	1 (7)
Missing	0	0
Objective response rate		
CR, n (%)	4 (57)	10 (71)
95% CI	18.41–90.10	41.90–91.61
Objective response (CR + PR), n (%)	6 (86)	12 (86)
95% CI	42.13–99.64	57.19–98.22

### Pharmacokinetics

The PK Population comprised 9 of the 11 patients treated in Part A; 2 patients were excluded from PK analyses due to extended IV belinostat infusion times (1.417 and 3.583 h, respectively) resulting in post-infusion profiles that were shifted in time by the additional infusion duration. Since no marked difference between the 2 belinostat dose levels was observed, and the treatments and sample collection times on the PK evaluation day (Cycle 1 Day 2) were identical, data at both dose levels were combined.

**Fig. 2:**
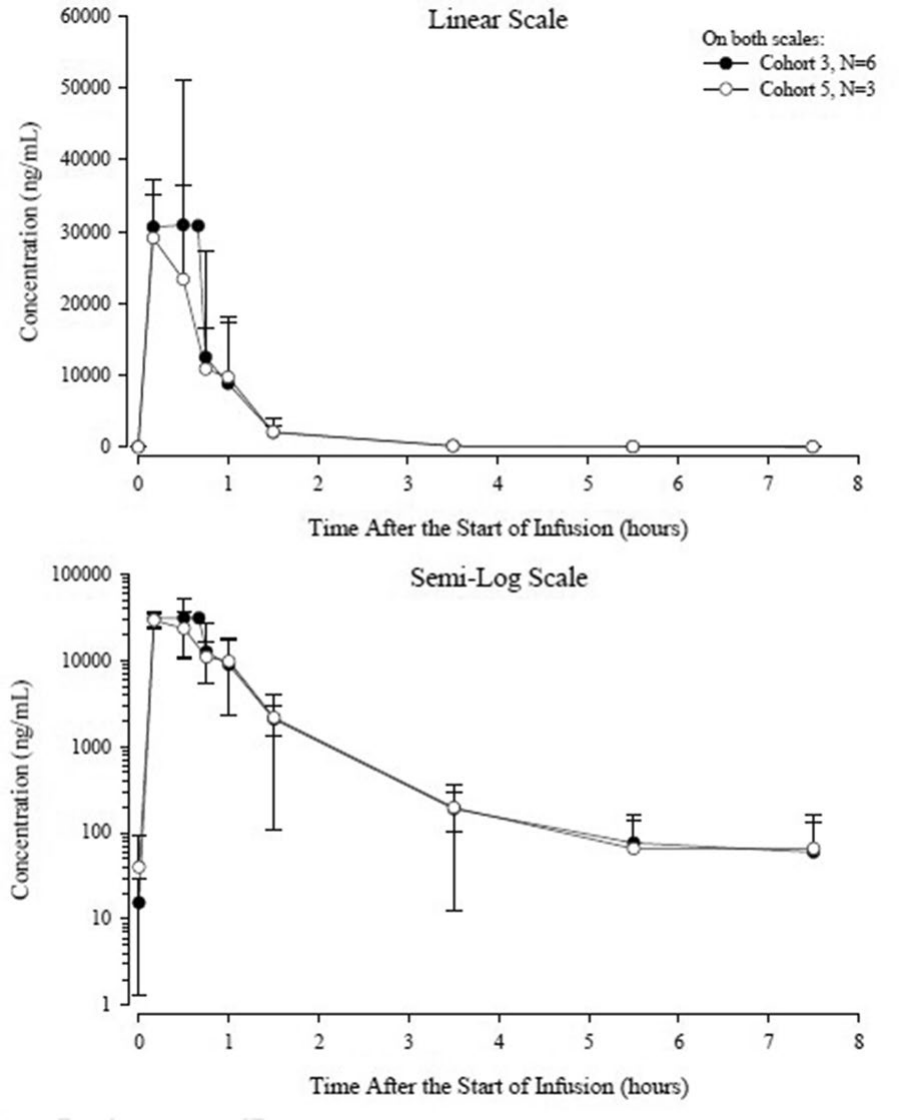
Mean ± SD plasma belinostat concentrations on cycle 1 day 2—PK population (N = 9)

Following daily 30-min IV infusions of 1000 mg/m^2^ belinostat for 2 days, the median time to maximum plasma concentration (T_max_) was observed at 0.5 h, ie, the approximate end of infusion (EOI). After EOI, plasma belinostat concentrations declined rapidly, followed by a relatively slower terminal phase. At the last collection time (7 h after EOI), plasma concentrations remained above the assay lower limit of quantitation (5 ng/mL) (Fig. 2). Belinostat exposure parameters (maximum plasma concentration [C_max_] 36,300 ng/mL and area under the curve [AUC_0-t_] 25,500 ng·hr/mL) were consistent with exposure parameters observed with single-agent that were published previously [28], indicating that the PK of belinostat was not affected by co-administration with CHOP (Table 5). Moderate interpatient variability in exposure was observed, with geometric coefficients of variance values ranging from 24.9% to 33.9% for C_max_ and AUC_0-t_. Belinostat glucuronide was the primary metabolite, with a mean metabolite-to-parent (M:P) AUC ratio of 12.5. A correlation analysis of PK and ORR indicated no significant difference in plasma concentrations for responding versus non-responding patients.

**Table 5:** Belinostat and metabolite plasma parameters—PK population (N = 9)

PK Parameters (units)	Belinostat	Belinostat Glucuronide	Belinostat Acid	Belinostat Amide	Methyl Belinostat	3-ASBA
	Mean ± Standard Deviation N = 9					
C_max_ (ng/mL)	36,300 ± 9320	163,000 ± 19,200	1030 ± 638	2850 ± 869	7030 ± 1270	2660 ± 1530
T_max_^a^ (hr)	0.500 (0.167, 1.10)	0.667 (0.550, .983)	1.60 (1.10, 3.68)	0.667 (0.500, 0.983)	1.10 (0.467, 1.68)	3.57 (3.50, 3.68)
AUC_0-t_ (ng^.^hr/mL)	25,500 ± 8980	472,000 ± 111,000	4760 ± 2790	10,600 ± 5010	25,200 ± 6870	13,000 ± 7060
M:P ratio	NA	12.5 ± 3.15	0.194 ± 0.079	0.514 ± 0.398	1.03 ± 0.451	0.579 ± 0.206

*AUC*_*0-t*_ area under the concentration time curve from time of administration to the last measurable concentration, estimated by the linear up/log down trapezoidal rule, *C*_*max*_ maximum plasma concentration, *M:P* metabolite:parent, *PK* pharmacokinetic(s), *T*_*max*_ time to C_max_

^a^Median (range)

## Discussion

The incorporation of novel agents into traditional cytotoxic regimens could be an opportunity to improve clinical benefits for patients with PTCL, both in the newly diagnosed and relapsed or refractory patient populations. The often dose prohibitive myelosuppression associated with traditional combination regimens (e.g., CHOP) has confounded efforts to integrate new agents into the treatment strategy, as the new agent must exhibit a unique mechanism of action that provides added efficacy without inducing further myelosuppressive effects [29, 30]. Several prior clinical studies incorporated additional drugs with CHOP or CHOP-like regimens. A Phase 2 study of denileukin diftitox combined with CHOP enrolled 49 patients and resulted in a CR rate of 55%, PR rate of 10% for an ORR of 65% [31]. Another Phase 2 study alternated pralatrexate with CEOP in patients with PTCL [32]. Of the 33 enrolled patients, 52% achieved a CR and 18% achieved a PR for an ORR of 70%. An additional Phase 1 study incorporated brentuximab vedotin with CHP for CD-30 expressing PTCL in which 26 patients were treated with a CR rate of 92% [33]. The majority of patients enrolled had systemic anaplastic large-cell lymphoma. Thus, several studies have incorporated novel agents into a CHOP or CHOP-like backbone with varying efficacy.

HDACi therapies are a particularly intriguing class of agents that have been recently studied in combination with standard first-line CHOP in PTCL [23, 24, 34]. The utility of HDACi in the combination setting is based on the typically low incidence of observed myelosuppression coupled with impressive clinical benefit. In a recently presented phase III trial of romidepsin plus CHOP chemotherapy versus CHOP chemotherapy alone, the addition of romidepsin to CHOP did not improve PFS, the primary endpoint of the study, and response rates and OS appeared similar with the combination [35]. It was noted that due to increased TEAEs with the addition of romidepsin, it hampered the ability to give the full 6 cycles of CHOP. In the pivotal study of belinostat monotherapy administered to a heavily pretreated population of patients with relapsed or refractory PTCL, the Grade 3/4 hematological toxicities common to CHOP therapy were reported at low incidences (ie, 7% thrombocytopenia, 6% neutropenia, 11% anemia) and the unique mechanism of action induced an ORR of 26% in a patient group that had already failed to achieve durable responses from currently available therapies [22]. This low myelosuppression and relatively high response induction suggested that Bel-CHOP therapy may represent a viable treatment strategy requiring further exploration.

## Conclusions

Results from the current study provided data to support this question and indicated that Bel-CHOP therapy was well tolerated and induced a high percentage of clinical responses when administered as first-line therapy in patients with newly diagnosed, previously untreated PTCL. Notably, the optimal dose of belinostat in the Bel-CHOP regimen was determined to be equivalent to the single-agent belinostat dose and schedule (1000 mg/m^2^ QD on Days 1–5), indicating that no additional toxicity was observed with the combination compared with belinostat monotherapy. Furthermore, the type and severity of AEs, including SAEs, was representative of the hematological (eg, anemia, neutropenia) and gastrointestinal (nausea, vomiting, constipation, diarrhea) toxicities that are often characteristic of cytotoxic therapies, and were often reported at similar incidence rates as historically reported with CHOP alone. The PK of belinostat further confirmed that the exposure of belinostat was not affected by co-administration with CHOP, with comparable C_max_ and AUC values as have been observed with belinostat monotherapy.

Notably the ORR contains higher patients with a CR (71%) MTD dose schedule of Bel-CHOP. As this study represented the first investigation of the Bel-CHOP regimen, progression-free-survival and overall survival were not studied. However, the promising response rate and safe profile of Bel-CHOP regimen requires further confirmatory study of this combination regimen.

## Data Availability

All data generated or analysed during this study are included in this published article.

## Abbreviations

AE: Adverse Event
AITL: Angioimmunoblastic T-cell lymphoma
ALCL: Anaplastic large-cell lymphoma
ANC: Absolute neutrophil count
ASCO: American Society of Clinical Oncology
AST and ALT: Alanine and aspartate aminotransferases
AUC: Area under the curve
Bel-CHOP: Belinostat combined with CHOP
CHOP: Cyclophosphamide, doxorubicin, vincristine, and prednisone
CR: Complete response
CTCAE: Common Terminology Criteria for Adverse Events
CTCL: Cutaneous T-cell lymphoma
DLT: Dose-limiting toxicities
DoR: Duration of response
ECG: Electrocardiograms
ECOG: Eastern Cooperative Oncology Group
EOI: End of Infusion
EOS: End of Study
G-CSF: Granulocyte colony-stimulating factor
HDACi: Histone deacetylase inhibitor
ICH: International Conference on Harmonization
MAD: Maximum administered dose
MTD: Maximum tolerated dose
NCI: National Cancer Institute
NK: Natural killer
ORR: Overall response rate
ORR: Overall response rate
OS: Overall survival
PD: Progressive disease
PFS: Progression-free survival
PK: Pharmacokinetics
PTCL: Peripheral T-cell lymphoma
PTCL-NOS: PTCL not otherwise specified
RP3D: Recommended Phase 3 dose
SAE: Serious Adverse event
TEAE: Treatment-emergent adverse event
US: United States
WHO: World Health Organization
